# Comparison of bronchoalveolar lavage fluid mNGS and sputum culture in the clinical application of severe pneumonia

**DOI:** 10.3389/fcimb.2025.1593818

**Published:** 2025-06-04

**Authors:** Na Gao, Jianwei Yin, Na Niu, Wendong Hao, Xiushan Chen, Tian Yang

**Affiliations:** ^1^ Department of Respiratory and Critical Care Medicine, The First Affiliated Hospital of Xi’an Jiaotong University, Yulin Hospital, Yulin, Shanxi, China; ^2^ Department of Allergy, The First Affiliated Hospital of Xi’an Jiaotong University, Yulin Hospital, Yulin, Shanxi, China; ^3^ Department of Respiratory and Critical Care Medicine, The First Affiliated Hospital of Xi’an Jiaotong University, Xi’an, Shanxi, China

**Keywords:** BALF mNGS, sputum culture, severe pneumonia, pathogen, treatment

## Abstract

**Objective:**

The purpose of this study is to compare the clinical application value of bronchoalveolar lavage fluid (BALF) mNGS and sputum culture in severe pulmonary infections, and to provide guidance for clinicians in selecting the appropriate testing method.

**Methods:**

This study collected 105 patients diagnosed with severe pneumonia, of which 55 patients who underwent BALF mNGS. We compared the pathogen detection rates, length of stay and mortality rate, treatment, and pathogen species between BALF mNGS group and sputum culture group.

**Results:**

The pathogen detection rate in BALF mNGS group was significantly higher than that in sputum culture group (*P*<0.0001). The length of hospital stay in the BALF mNGS group was shorter than that in the sputum group (*P*=0.0093). There was no statistically significant difference in mortality rate between the two groups (*P*=0.26). However, BALF mNGS group had a lower antibiotic usage rate than the sputum culture group (*P*=0.0491). According to the mNGS results, initial antipathogenic treatment was modified in 67.27% of patients. In BALF mNGS group, the main pathogens detected included *Mycoplasma pneumoniae*, *Mycobacterium tuberculosis* (MTB) and *Haemophilus influenzae* (HI). The sputum culture group mainly included: MTB, HI.

**Conclusion:**

BALF mNGS effectively and rapidly identifies pathogens, helping doctors quickly diagnose severe pneumonia pathogens. Combined with the patient’s medical history, laboratory results and imaging, clinical doctors can adjust the patient’s treatment plan in time. This has potential advantages in improving the cure rate of severe pneumonia patients, reducing the length of hospital stay, and improving the prognosis.

## Introduction

The mortality rate of severe pneumonia is relatively high, and the rapid and accurate identification of pathogens is crucial for clinical treatment (Nair and Niederman, 2021). Although traditional pathogen culture is considered the gold standard for diagnosis, it has some limitations, including low positive rates, long duration, and difficulty in detecting atypical pathogens (such as viruses and mycoplasmas, etc.) (Miao et al., 2018; Gu et al., 2019). The results show that the detection rate of pathogenic bacteria in conventional culture is very low in pulmonary infections, especially in patients who have received antibiotic treatment (Mandell et al., 2007).

Metagenomic next-generation sequencing (mNGS) does not rely on traditional microbial culture; it allows unbiased extraction of all nucleic acids from samples for high-throughput sequencing. Through bioinformatics analysis, human sequences are removed, and the data is compared with pathogen databases to obtain information on the species of suspected pathogenic microorganisms (Goldberg et al., 2015). This technology is renowned for its speed, high sensitivity, and broad coverage, and it has shown significant clinical application potential, especially in detecting rare and atypical pathogens (Hilton et al., 2016; Parize et al., 2017; Somasekar et al., 2017).

Sputum culture is a traditional detection method, although its culture rate is low and the types are limited, it is easy to obtain and safe. It is the most common sample type used for diagnosing respiratory diseases and is widely accepted and recognized by more patients and their families. BALF needs to be obtained under bronchoscopy, which carries certain risks and the culture rate is not very high (Carugati et al., 2018; Liu et al., 2022). It cannot be understood and accepted by most patients, nor can it meet clinical needs. Compared with BALF culture, sputum culture is easy to obtain and safe, and is widely used in clinical practice. A study on infectious diseases mentioned that there was no significant difference in the positive rate between sputum culture and BALF culture (Miao et al., 2018). Due to respiratory cultivation process is time-consuming, which limits their ability to provide support for rapid and accurate diagnosis of severe pneumonia, thereby affecting diagnosis and treatment and mortality risk. Because BALF is more representative of pathogens at the site of infection than sputum, BALF mNGS has more advantages than sputum mNGS. The patients were willing to undergo bronchoscopic bronchoalveolar lavage for mNGS testing, but not culture. However, the clinical application value of BALF mNGS in patients with severe pneumonia is not yet clear. The objective of this study is to evaluate and compare the performance of BALF mNGS and sputum culture in the diagnosis of severe pneumonia, in order to assist in the clinical diagnosis and treatment of severe pneumonia.

## Methods

### Patients and study design

This was a retrospective study that recruited 105 patients from July 2023 and August 2024, at Yulin Hospital, the First Affiliated Hospital of Xi’an Jiaotong University, in Yulin. All patients with severe pneumonia were diagnosed and classified according to the guidelines for diagnosis and treatment of CAP in Chinese Adults(2016 edition)released by the Chinese Thoracic Society (2016). The inclusion criteria are as follows: 1. Age ≥ 18 years old; 2. Those who meet one of the following primary criteria or ≥ three secondary criteria can be diagnosed with severe pneumonia. Main criteria: (1) Mechanical ventilation therapy with tracheal intubation is required; (2) Septic shock still requires vasoactive drug treatment after active fluid resuscitation. Secondary criteria: (1) Respiratory rate ≥ 30 times/min; (2) Oxygenation index ≤ 250mmHg (1mmHg=0.133kPa); (3) Multiple lung lobes infiltration; (4) Consciousness disorders and/or orientation disorders; (5) Blood urea nitrogen ≥ 7.14mmol/L; (6) Systolic pressure < 90mmHg requires active fluid resuscitation. Exclusion criteria: asthma, allergic pneumonia, radiation pneumonitis, and interstitial lung disease. Among the 105 patients we collected, 55 patients or their families agreed to undergo BALF mNGS; The remaining 50 patients underwent routine pathogen culture, including sputum production or aspiration. In our study, the identification of the pathogen was ultimately determined through a comprehensive analysis of the patient’s condition, laboratory test results, and imaging data by clinical doctors.

### Sample processing and sequencing

With the consent of the patient or their family, after admission, the most significant or rapidly progressing site of the lesion was selected through imaging examination for lavage. The top of the bronchoscope was embedded in the target lavage lung segment or sub segment bronchial opening position. Using a syringe, 0.9% room temperature sterile saline was rapidly injected through the bronchoscope operating hole in batches, with 20–50 ml injected each time, and the total amount controlled at 60–120 ml (The Respiratory Critical Care Medicine Group of the Respiratory Medicine Branch of the Chinese Medical Association and Critical Care Medicine Working Committee of the Respiratory Medicine Branch of the Chinese Medical Association, 2020). Recycled samples were sealed in polypropylene recycling bottles. The 20 milliliters in the first tube may contain pathogenic bacteria from non diseased areas, affecting the test results. Therefore, the remaining samples were collected for testing. The samples were stored at 4 °C and sent to the sequencing center for testing within 24 hours (Dian Diagnostics, China). Because the collection volume of BALF is limited; The results of BALF mNGS were earlier than those of BALF culture; The positive rate of BALF culture is not high, and the types that can be detected are limited. Most patients are unwilling to spend more money on BALF culture at the same time.

Firstly, a biological sample homogenizer is used to break down the cell walls of microorganisms in BALF samples, in order to improve the efficiency of subsequent nucleic acid extraction. 1.2 ml sample was separated into a shaking tube for homogenization treatment. Subsequently, DNA and RNA were extracted from the sample using a nucleic acid extraction kit (magnetic bead method) and a fully automated nucleic acid extractor. For RNA samples, reverse transcription is performed to generate cDNA. We used a standard library construction process for nucleic acid fragmentation, end repair, A-tail addition, and index ligation. Targeted multiplex amplification was performed on bacterial 16S rRNA gene regions, fungal ITS regions, as well as virus and parasite specific gene fragments for library construction. The amplicons were subjected to high-throughput sequencing using third-generation nanopores. Next, Fastp software removed adapter sequences and filtered out low-quality sequencing reads with base quality values below 20 (Q20). Subsequently, Burrows Wheeler Aligner (BWA) aligned the cleaned sequencing data to human genome databases (hg38, YH genome, T2T-CHM13 genome) and extracted sequencing readings that were not aligned with human readings. Finally, BWA was used to compare the sequencing reads obtained after removing human reads with NCBI GenBank and internal pathogenic microorganism genome databases. In order to identify suspected pathogenic microorganisms, advanced data analysis was performed using internal scripts, including classification annotation, genome coverage, depth calculation, and abundance calculation. Excess specimens should be disinfected appropriately to ensure that the pathogens present are inactivated. The processed waste should meet environmental protection requirements and avoid polluting the environment.

Due to the risks associated with bronchoscopy and the high cost of BALF mNGS, some patients refuse BALF mNGS. For the 50 patients who did not agree to undergo BALF mNGS testing, medical staff instructed them to rinse their mouths, take deep breaths, and cough up phlegm forcefully upon admission; If some patients were unable to cough up sputum on their own, medical staff used suction tubes for collection. The collected sputum specimens were sent to the laboratory for testing within 1 hour. The number of epithelial cells and white blood cells were evaluated by Gram staining to determine whether they were qualified lower respiratory tract specimens (i.e., under a 10x microscope, the average number of squamous epithelial cells per field of view is less than 10, white blood cells more than 25, or white blood cells: squamous epithelial cells>2.5) (Carroll et al., 2019). Subsequently, a certain amount of sputum specimens were inoculated onto different bacterial, fungal, and Mycobacterium tuberculosis culture media, incubated at a constant temperature of 35-37°C, and the culture results were observed and recorded. The laboratory identifies pathogens through smear microscopy, colony characteristics, biochemical tests, mass spectrometry techniques, and other methods. According to standard operating procedures, the effectiveness of various antibiotics can be determined using drug sensitive paper diffusion method or microdilution method.

### Statistical analysis

All data were analyzed and processed using Prism 6.0 software (GraphPad, La Jolla, CA). Continuous variables are presented as medians and categorical variables as counts and percentages. Statistical analysis was performed using Mann Whitney U test or Chi-square test for differences between groups. Statistical significance was considered to be *P*< 0.05.

## Results

### Demographics and baseline characteristics

From July 2023 to August 2024, a total of 105 patients diagnosed with severe pneumonia were included in this study, including 55 cases in the BALF mNGS group and 50 cases in the sputum culture group. Demographic characteristics and clinical symptoms are shown in **Table 1**.The clinical characteristics of all participants included the gender, age, initial symptom, comorbidity, treatment and clinical parameters. The median ages of patients in the BALF mNGS group and the sputum culture group were 61 years and 63 years, with male proportions of 58.18% and 28%, respectively. The two queues are similar in the median age and gender composition. The most common initial symptoms in BALF mNGS patients were cough accompanied by dyspnea (12/55, 21.82%), followed by fever accompanied by cough (10/55, 18.18%), dyspnea (9/55, 16.36%), and fever accompanied by cough and dyspnea (9/55, 16.36%). Although the most common initial symptoms among patients in the sputum culture group were fever accompanied by cough (10/50, 20.00%), followed by cough accompanied by dyspnea (9/50, 18.00%), dyspnea (7/50, 14.00%), fever accompanied by cough and dyspnea (9/55, 16.36%), there was no statistically significant difference in symptoms between the two groups. The most common complication of the two groups was COPD (13/55, 23.64% vs. 10/50, 20.00%), followed by diabetes (8/55, 14.55% vs. 7/50, 14.00%). The median length of hospital stay for patients in the BALF mNGS group (9 vs. 11, *P*=0.0080) was less than that of the sputum culture group. In BALF mNGS group, the proportion of patients receiving antibiotic treatment, antiviral treatment, antifungal treatment and referred to infectious disease (tuberculosis) hospital were 78.18%, 16.36%, 7.27% and 18.18%, respectively. In contrast, the corresponding proportions in the sputum culture group were 92.00%, 26.00%, 4.00%, and 10.00%. There was a significant difference in antibiotic use between the two groups, with statistical significance (*P*=0.0491).

**Table 1:** Demographic and clinical characteristics of patients with severe pneumonia.

Parameter (median or n [%])	Total (N=105)	BALF mNGS (N=55)	Sputum culture (N=50)	*P* value
Age (years)	62 (18-84)	61 (18-81)	63 (18-84)	0.07
Gender (male)	60 (57.14)	32 (58.18)	28 (56.00)	0.8
Clinical symptoms				
Fever	4 (3.80)	1 (1.82)	3 (6.00)	NS
Cough	10 (9.52)	6 (10.91)	4 (8.00)	NS
Dyspnea	16 (15.23)	9 (16.36)	7 (14.00)	NS
Chest tightness	3 (2.86)	1 (1.82)	2 (4.00)	NS
Fever and cough	20 (19.05)	10 (18.18)	10 (20.00)	NS
Fever and dyspnea	7 (6.67)	3 (5.45)	4 (8.00)	NS
Cough and dyspnea	21 (20.00)	12 (21.82)	9 (18.00)	NS
Fever, cough and dyspnea	16 (15.23)	9 (16.36)	7 (14.00)	NS
Cough and chest tightness	5 (4.76)	2 (3.64)	3 (6.00)	NS
Fever and chest tightness	3 (2.86)	2 (3.64)	1 (2.00)	NS
Clinical indicators				
length of stay (days)	10 (4-28)	9 (4-20)	11 (4-28)	0.0080
Die	4 (3.80)	1 (1.82)	3 (6.00)	0.26
Comorbidity				
Diabetes	15 (14.29)	8 (14.55)	7 (14.00)	NS
Hypertension	11 (10.48)	6 (10.91)	5 (10.00)	NS
Coronary heart disease	9 (8.57)	6 (10.91)	3 (6.00)	NS
Stroke	3 (2.86)	2 (3.64)	1 (2.00)	NS
Malignant tumor	3 (2.86)	2 (3.64)	1 (2.00)	NS
COPD	23 (21.90)	13 (23.64)	10 (20.00)	NS
Final treatment				
Antibiotic	89 (84.76)	43 (78.18)	46 (92.00)	0.0491
Antiviral	22 (20.95)	9 (16.36)	13 (26.00)	NS
Antifungal	6 (5.71)	4 (7.27)	2 (4.00)	NS
Referral to an infectious disease hospital	15 (14.29)	10 (18.18)	5 (10.00)	NS

COPD, chronic obstructive pulmonary disease.

### Pathogen detection results in severe pneumonia

In this study, we conducted pathogen testing on 105 patients with severe pneumonia. Among them, 55 cases had mNGS with BALF samples, and 50 cases had pathogen culture with sputum samples (**Table 2**). The comprehensive evaluation results of clinical doctors showed that the detection rate of mNGS in BALF was 78.18% (43/55), significantly higher than the detection rate of sputum culture, which was 30.00% (15/50), *P*<0.0001. BALF mNGS identification of pathogenic pathogens takes 6–24 hours, while general bacterial sputum culture takes 2–5 days, anaerobic or special bacteria takes 5–7 days, and tuberculosis bacteria takes 4–8 weeks.

**Table 2:** Pathogen detection results in severe pneumonia.

Groups (n [%])	Pathogen detection results		Total	*P* value
	+	-		
BALF mNGS	43 (78.18)	12 (21.82)	55	< 0.0001
Sputum culture	15 (30.00)	35 (70.00)	50	

### Mixed infections and pathogens Detected by BALF mNGS

**Figure 1:**
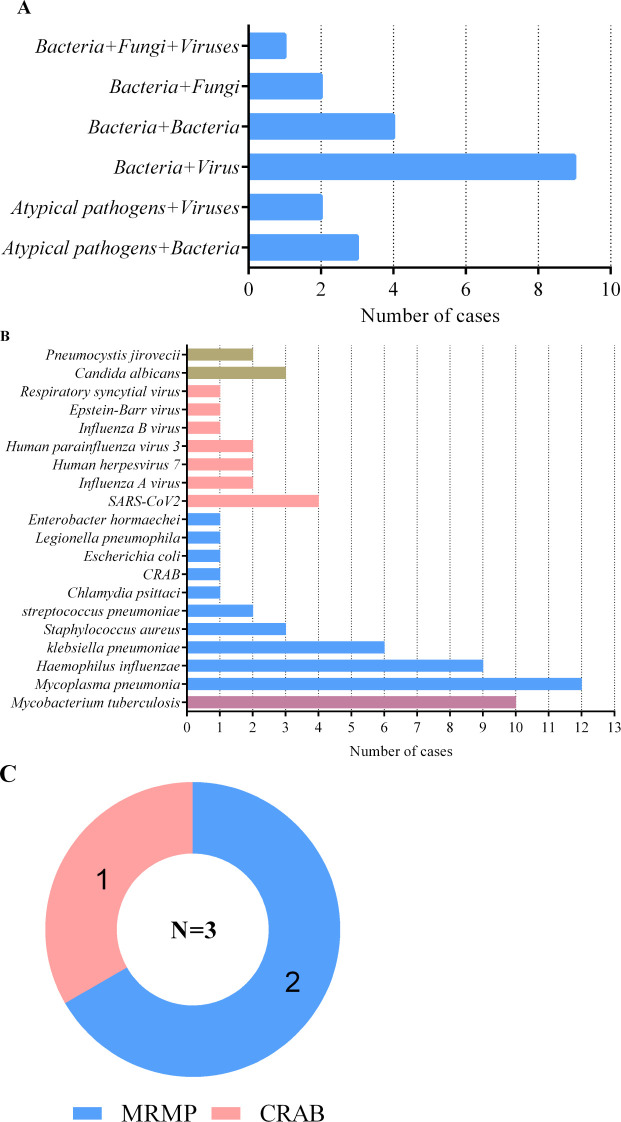
Mixed infections and pathogens identified by BALF mNGS in 55 severe pneumonia patients. **(A)** number of severe pneumonia patients with mixed infections; **(B)** number of severe pneumonia patients infected with pathogens; **(C)** number of drug-resistant bacteria. MRMP, macrolide-resistant mycoplasma pneumoniae; CRAB, carbapenem-resistant acinetobacter baumannii.

Due to the complexity and variability of severe pneumonia, as well as the resulting weakened immune system, there is a situation of mixed infection among patients with severe pneumonia. The number of pathogens detected by mNGS is shown in **Figure 1**. In severe pneumonia, viruses and bacteria are the most common co-infection pathogens observed. Among 55 patients with severe pneumonia, mNGS identified 9 (16.36%) cases of viral–bacterial co-infections and 4 (7.27%) cases of bacterial–bacterial co-infections (**Figure 1A**). The first six pathogens detected by mNGS included *M. pneumonia*, MTB, HI, *Klebsiella pneumoniae*, SARS-CoV2, *Staphylococcus aureus and Candida albicans* (**Figure 1B**). It is worth noting that MTB was detected in 10 patients with severe pneumonia. We should pay more attention to whether severe pneumonia is accompanied by tuberculosis infection. All 10 tuberculosis patients were referred to infectious disease (tuberculosis) hospital and received anti-tuberculosis treatment. In addition, BALF mNGS detected 3 cases of drug-resistant bacteria, 2 cases of macrolide resistant *M. pneumoniae*, and 1 case of carbapenem resistant *Acinetobacter baumannii* (**Figure 1C**).

### Pathogens detected by sputum culture

**Figure 2:**
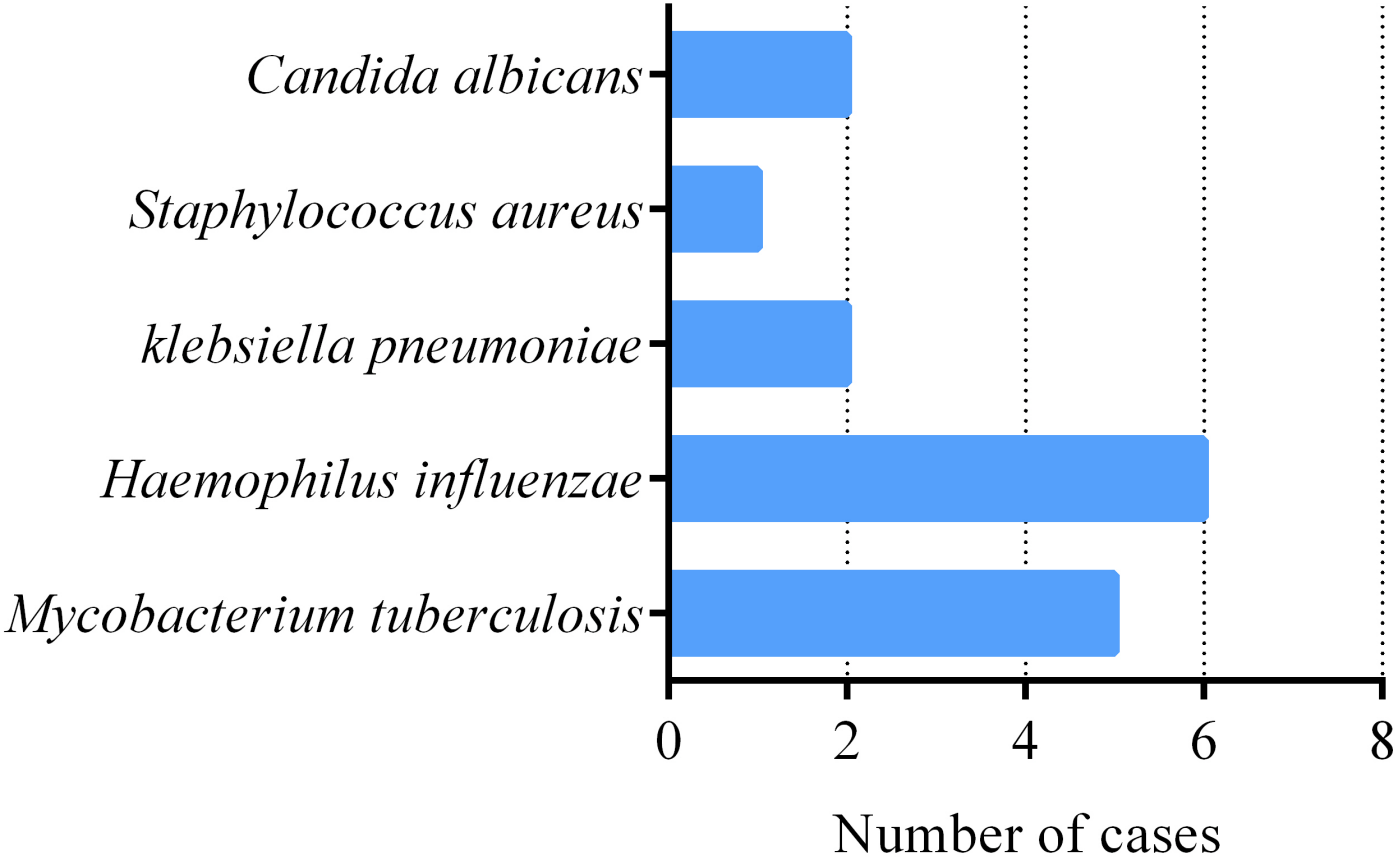
Pathogens Detected by sputum culture in 50 severe pneumonia patients. number of severe pneumonia patients with pathogens.

Among 50 patients with severe pneumonia, the number of pathogenic bacteria detected through sputum culture was relatively small. The top three pathogens were, in order, HI, MTB, *K. pneumoniae*, and *C. albicans* (**Figure 2**). Surprisingly, the detection rate of MTB was second only to HI, which also suggests that we should be particularly vigilant about the possibility of tuberculosis infection in patients with severe pneumonia. All 5 tuberculosis patients were referred to infectious disease (tuberculosis) hospital and received anti-tuberculosis treatment. For patients whose sputum culture results do not detect pathogenic bacteria, respiratory and critical care physicians will comprehensively consider factors such as the patient’s clinical history, laboratory test results, imaging data, and local disease prevalence, and selectively use antibiotics, antiviral drugs, or antifungal drugs for treatment based on clinical experience.

### Impact of BALF mNGS on antipathogenic treatment of severe pneumonia Patients

Records of treatment were retrieved from 55 severe pneumonia patients. According to the mNGS results, initial antipathogenic treatment was modified in 67.27% of patients; 40% of patients had one or two antipathogenic drug removed; 10.91% had the antimicrobial spectrum reduced, and 16.36% had one or two antipathogenic drug increased (**Table 3**). For patients in whom initial antimicrobial therapy was not modified after receiving mNGS results, the reason was that the antibacterial spectrum of the therapeutic drug was consistent with the mNGS results, and the treatment evaluation was effective.

**Table 3:** Impact of BALF mNGS on antipathogenic treatment of severe pneumonia Patients.

Modifications (n [%])	BALF mNGS (N=55)
Remove 1 drug	18 (32.73)
Remove 2 drugs	4 (7.27)
Reduce antimicrobial spectrum	6 (10.91)
Add 1 drug	6 (10.91)
Add 2 drugs	3 (5.45)
No change	18 (32.73)

Remove 1 (or2) drug, the number of antipathogenic drug types reduced by 1 (or 2) after the report of BALF mNGS results; Add 1 (or 2) drug, the number of antipathogenic drug types increased by 1 (or 2) after the report of BALF mNGS results.

## Discussion

Severe pneumonia is a serious respiratory disease with a variety of complex pathogenic microorganisms. It progresses rapidly, has a high mortality rate, and survivors often have poor outcomes, such as changes in lung function, decline in mental and cognitive abilities, weight loss, and reduced activity function (Bein et al., 2018; Sangla et al., 2020). Early identification of the infection type in severe pneumonia is crucial for its treatment. This helps to shorten hospital stays and improve patient prognosis.

Traditional pathogen diagnosis methods, such as culture and polymerase chain reaction (PCR), have drawbacks like low positivity rates, limited detectable species, and long processing times (Gu et al., 2019). Our study revealed that the pathogen detection rate in sputum cultures for severe pneumonia patients was only 30.00% (15 out of 50), far from meeting clinical needs. Additionally, the sensitivity of traditional cultures was affected by the use of antibiotics in the clinical environment. Research by Malik & Bhattacharyya (Malik and Bhattacharyya, 2019) and Frost et al (Frost et al., 2019). confirmed that the use of broad-spectrum antibiotics reduces the sensitivity of traditional cultures. Kottmann et al (Kottmann et al., 2011). reported that the optimal time for BALF culture was within the first three days of initiating broad-spectrum antibiotic treatment, after which the positivity rate significantly decreases. In reality, many severe pneumonia patients had already received antibiotic treatment before hospital admission, further lowering the positivity rate of sputum cultures and posing challenges for the diagnosis and treatment of severe pneumonia.

Previous studies had shown a high consistency between mNGS and traditional microbiological testing (Langelier et al., 2018; Li et al., 2018; Pan et al., 2019; Qi et al., 2019; Xie et al., 2019). Additionally, another study indicated that mNGS was less affected by antibiotic exposure compared to conventional culture (Miao et al., 2018). These findings suggested that applying BALF mNGS for pathogen diagnosis in severe pneumonia is promising and warrants further exploration.

In respiratory tract infections, BALF is more representative of pathogens at the site of infection compared to sputum or throat swabs. Our study confirmed that the detection rate of BALF mNGS in severe pneumonia patients (78.18% (43/55)) was significantly higher than that of sputum culture, which aids clinical decision-making.

Moreover, recent studies supported the advantages of mNGS in detecting mixed infections, especially for difficult-to-culture and time-consuming pathogens such as atypical pathogens, viruses, MTB, anaerobic bacteria, and fungi (Parize et al., 2017; Pan et al., 2019). Our research demonstrated that viral and bacterial co-infections are the most common mixed infection patterns observed in severe pneumonia patients. A recent nationwide surveillance study also suggested that the rate of virus-bacteria co-infection is higher than that of virus-virus and bacteria-bacteria co-infections in severe community acquired pneumonia (SCAP) patients (Liu et al., 2023). Viral infection may cause respiratory damage, further promoting bacterial attachment and growth, while bacterial infection may increase the risk of viral infection by altering the difficulty of virus transmission and infection within the respiratory system (Bosch et al., 2013; Bakaletz, 2017; Cawcutt and Kalil, 2017). Compared to single pathogen (virus or bacteria) infections, mixed infections have more complex clinical manifestations, more severe inflammatory reactions, greater treatment difficulty. These virus-bacteria co-infections lead to worsened clinical outcomes, including prolonged hospital stays and increased mortality (Cawcutt and Kalil, 2017). Therefore, early effective identification of mixed pathogens in BALF using mNGS can improve patient prognosis.

In our study, the pathogens detected by BALF mNGS mainly included *M. pneumonia*, MTB, HI, *K. pneumoniae*, SARS-CoV2, *S. aureus* and *C. albicans*. Apart from atypical pathogens and viruses, the remaining major pathogens were largely similar between the two groups of patients.

Previously, *M. pneumoniae* was often considered the primary pathogen for pediatric respiratory infections, while adult infections were typically regarded as mild and self-limiting. However, we found that the main pathogen in severe pneumonia patients in the BALF mNGS group was *M. pneumoniae*, with five cases involving co-infections with other pathogens. Although this finding is inconsistent with previous research on pathogens causing severe pneumonia in adults, it is not surprising. As noted by Garzoni et al (Garzoni et al., 2024). and Zayet et al (Zayet and Klopfenstein, 2024). in their letters, since November 2023, there has been an unexpected increase in severe pneumonia cases caused by *M. pneumoniae* in adults. Zayet et al (Zayet and Klopfenstein, 2024). emphasized that in the current epidemiological context, it was crucial to abandon the outdated view that this disease was usually mild in adults and did not require specific microbiological diagnostic tests. This highlights the significant role of BALF mNGS in identifying underappreciated or atypical pathogens in severe pneumonia cases.

Like atypical pathogens, viruses were difficult to culture and detect, but they can also be effectively screened by mNGS (Qian et al., 2021; Bassi et al., 2022; Fourgeaud et al., 2024). In our collected data, 13 cases of viral infections were detected in severe pneumonia patients through BALF mNGS, with 12 of these cases co-infected with other pathogens leading to severe pneumonia. This further demonstrates that in the diagnosis of severe pneumonia caused by viral infection or co-infection with viruses, BALF mNGS has a clear advantage over traditional pathogen culturing methods.

It is noteworthy that we found MTB accounted for a high proportion in both sputum culture and BALF mNGS groups. However, according to our statistics, most patients had already been discharged when the results of the tuberculosis culture were available. This indicates that the culture method has a lag, which cannot provide timely and effective diagnostic evidence. For severe pneumonia patients, early identification of pathogens is crucial. Therefore, although sputum culture can effectively identify MTB, it is still at a disadvantage compared to BALF mNGS.

Our research indicates that BALF mNGS has a high detection rate for bacteria and fungi, and can identify various types of microorganisms, providing valuable information for clinical diagnosis. Additionally, BALF mNGS can detect whether pathogenic bacteria contain resistance gene targets. In our data, we identified three cases of resistant bacteria, including two cases of *M. pneumoniae* with positive 23S rRNA V region genes resistant to macrolides, and one case of *A. baumannii* with a positive OXA-23 gene resistant to carbapenems. Therefore, clinical doctors chose Moxifloxacin for the treatment of MRMP and Cefoperazone Sodium and Sulbactam Sodium combined with Tigecycline for the treatment of CRAB. These three patients had a better prognosis due to appropriate medication. Although the number of cases is relatively small, it also indicates that early detection of mNGS can reduce the risk of death in patients with drug-resistant bacterial infections. These results of drug resistance gene testing provide crucial references for clinical drug selection and management.

In this study, 67.27% of severe pneumonia patients in the BALF mNGS group had their initial antimicrobial therapy modified after receiving the mNGS results. Similarly, another study on infectious diseases found that the majority of infection diagnosis results were corrected based on mNGS test results (61.4%), while most empirical antibiotic treatment regimens were not applicable to detected pathogens (58.6%) (Miao et al., 2018). Related to this, we observed that the usage rate of antibiotics in the final treatment plans of patients in the BALF mNGS group was lower than that in the sputum culture group (78.18% vs. 92.00%, *P*=0.0491). Additionally, the length of hospital stay for patients in the BALF mNGS group was significantly shorter compared to the sputum culture group, although there was no statistically significant difference in mortality between the two groups. This may be related to the small sample size of this study. In summary, these findings indicate that BALF mNGS has significant application value in the diagnosis and treatment of severe pneumonia and can assist in changing patient clinical outcomes. However, this study has limitations. First, it is a single-center retrospective study; second, the sample size is small; Third, the interpretation of mNGS results cannot be accomplished by a single role or step, and collaborating with bioinformatics experts would be more ideal. Therefore, inherent biases are inevitable.

## Conclusion

Although BALF mNGS has many advantages over sputum culture, there are also some limitations. For example, how to choose an appropriate quantitative threshold for mNGS results to distinguish between colonization and infection still needs further exploration. Therefore, the etiological diagnosis of severe pneumonia should be based on a comprehensive consideration of clinical history, imaging, and microbiological evidence, rather than solely relying on BALF mNGS. Although the cost of BALF mNGS is relatively high, dynamic detection of BALF mNGS in patients with severe pneumonia throughout the entire disease process will help to investigate its potential application value in predicting the treatment efficacy and clinical outcomes of severe pneumonia.

## Data Availability

The original contributions presented in the study are included in the article/supplementary material. Further inquiries can be directed to the corresponding author.
